# miR-23b-3p suppressing PGC1α promotes proliferation through reprogramming metabolism in osteosarcoma

**DOI:** 10.1038/s41419-019-1614-1

**Published:** 2019-05-16

**Authors:** Ran Zhu, Xinpan Li, Yanhong Ma

**Affiliations:** 1 Department of Rehabilitation Medicine, Shanghai Jiao Tong University Affiliated Sixth People’s Hospital, Shanghai, China; 2 Department of Rehabilitation Medicine, Putuo People’s Hospital Affiliated to Tongji University, Shanghai, China

**Keywords:** Biologics, Bone cancer

## Abstract

Metabolic shift from oxidative phosphorylation (OXPHOS) to glycolysis is a hallmark of osteosarcoma (OS). However, the mechanisms of the metabolic switch have not been completely elucidated. Here we reported that the miR-23b-3p was significantly upregulated in OS cells. Functional studies suggested that knockdown of miR-23b-3p could inhibit OS cell proliferation in vitro or in vivo. In addition, suppression of miR-23b-3p could lead to upregulation of OXPHOS and suppression of glycolysis. Mechanistically, miR-23b-3p promoted OS cell proliferation and inhibited OXPHOS in OS, at least in part, by directly targeting peroxisome proliferator-activated receptor gamma coactivator-1 alpha (PGC1α) and inhibiting its expression. Our data highlights important roles of miR-23b-3p and PGC1α in glucose metabolism reprogram of OS. The suppression of miR-23b-3p may provide effective therapeutic strategies for the treatment of OS.

## Introduction

Osteosarcoma (OS) is a common malignant bone tumor and is the main cause of tumor-related deaths in children and adolescents^1^. Over the past 30 years, the development of chemotherapeutics and surgical operation have significantly improved the outcomes of OS patients^2^. However, still 30 – 40% of patients with OS develop recurrent or metastatic diseases, which results in poor outcomes^3^. Therefore, it is critical to identify the underlying mechanisms for OS development and progression, and explore new prognostic biomarkers as well as therapeutic targets for more effective therapeutic strategies.

MicroRNAs (miRNAs) are a kind of small non-coding RNA molecules (21–23 nucleotides), which regulate gene expression by binding to the 3′-untranslated regions (3′-UTR) of target mRNA and promoting target mRNA degradation or translational inhibition^4^. Previous studies identified that miRNAs could act as oncogenes or cancer inhibitors in various tumors and regulated a wide range of important tumor cell processes including cell proliferation, metastasis, apoptosis, and metabolic reprogramming^5–7^. The miR-23b-3p belongs to the miR-23b/27b/24–1 cluster and has been reported to function as an onco-miR in different cancers including glioma, gastric cancer, and breast cancer^8–10^. However, the functions and mechanisms of miR-23b-3p in OS have not been previously reported.

Ongoing studies have revealed that suppression of oxidative phosphorylation (OXPHOS) along with enhanced glycolysis, which is also called the Warburg effect, lead to chemoresistance, proliferation, and metastasis of cancer cells^11^. Peroxisome proliferator-activated receptor gamma coactivator-1 alpha (PGC1α) is a multi-functional transcriptional coactivator, which ensures maintenance of mitochondrial homeostasis and controls oxidative metabolism^12^. It has been reported that overexpression of PGC1α could lead to enhancement of mitochondrial OXPHOS and reduction of aerobic glycolysis, which is also termed anti-Warburg effect, in several types of tumors^13,14^. However, whether PGC1α is taken part in the glucose metabolism reprogram in OS remains unclear.

Here we demonstrated that miR-23b-3p is upregulated in OS cells. miR-23b-3p suppressed OXPHOS and promoted aerobic glycolysis in OS, resulting in the enhancement of OS cell proliferation. Furthermore, we identified potential target genes of miR-23b-3p and found that miR-23b-3p inhibited OS cell OXPHOS and growth, at least in part, by directly targeting PGC1α and inhibiting its expression.

## Results

### MiR-23b-3p is upregulated in OS cell lines and promotes the proliferation of OS cells

**Fig. 1:**
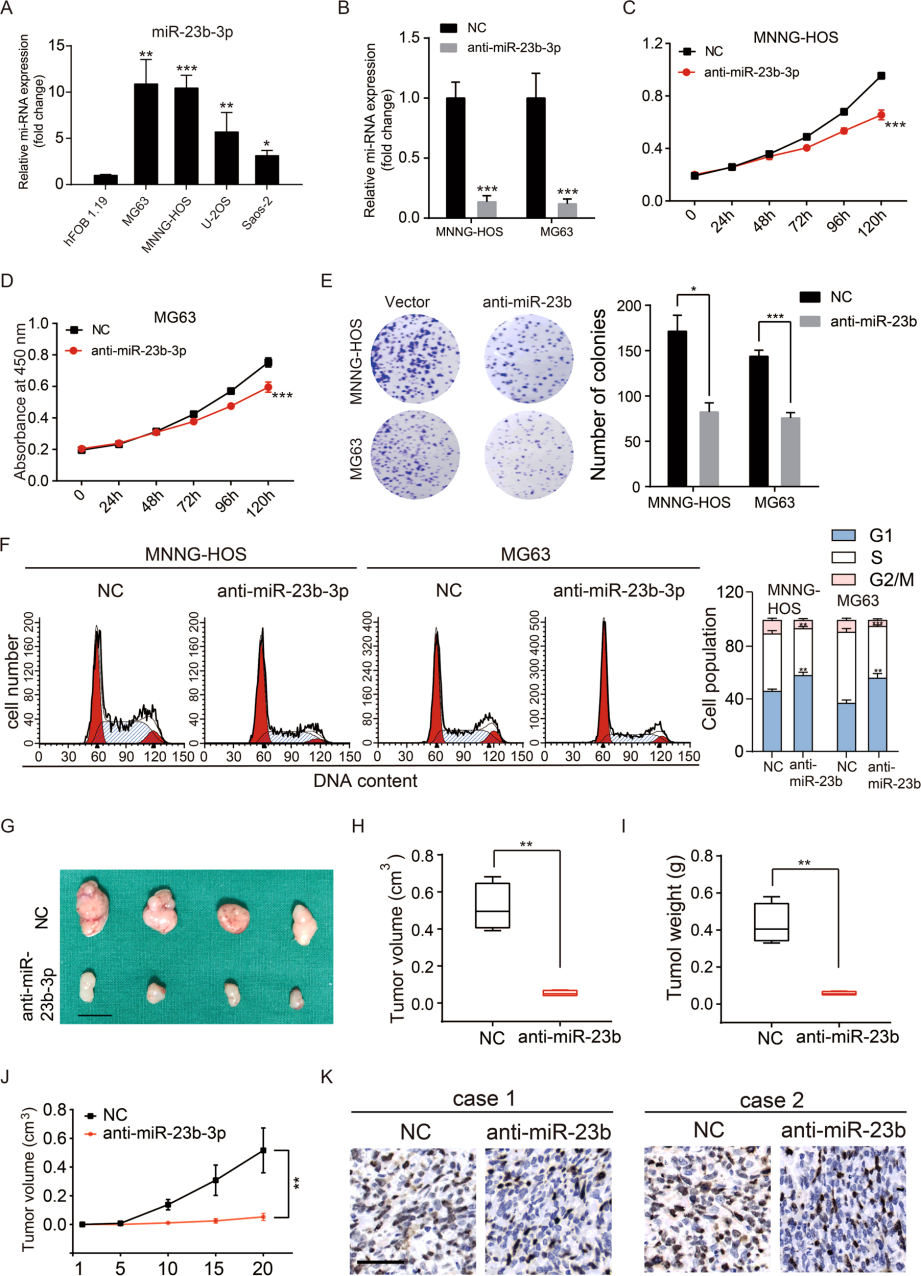
miR-23b-3p promoted OS proliferation in vitro and in vivo. **a** The expression patterns of miR-23b-3p in normal osteoblast cell line (hFOB1.19) and OS cell lines (MNNG-HOS, U-2OS, Saos-2, and MG63) by qRT-PCR. *p < 0.05, **p < 0.01, ***p < 0.001. **b** Knockdown efficacy of miR-23b-3p in wild OS cells (MNNG-HOS and *p < 0.05, **p < 0.01, ***p < 0.001. MG63) was determined by qRT-PCR, ***p < 0.001. **c**, **d**. Knockdown of miR-23b-3p inhibited MNNG-HOS and MG63 cell proliferation using the cell counting kit (CCK)-8 assay. Values are means ± SD, ***p *<* 0.001. **e** Downregulation of miR-23b-3p suppressed OS cell (MNNG-HOS and MG63 cells) proliferation using colony formation assay. Values are means ± SD, *p < 0.05, ***p < 0.001. **f** Knockdown of miR-23b-3p led to G1-phase cell cycle arrest and decreased S-phase cell population through using cell cycle analysis. Values are means ± SD, **p < 0.05, ***p < 0.001. **g** Morphologic characteristics of excised tumors from nude mice in MNNG-HOS/control group and MNNG-HOS/miR-23b-3p knockdown group (*n* = 4). Scale bars = 1 cm. **h** Tumor weight in miR-23b-3p overexpression group was reduced compared with control group (*n* = 4), **p < 0.01. **i** Tumor volume in miR-23b-3p overexpression group was reduced compared with control group (*n* = 4), **p < 0.01. **j** Tumor volumes were measured with calipers every 5 days. The growth rate in miR-23b-3p knockdown group was significantly slower than that in control group (*n* = 4), **p < 0.01. **k** Representative images of Ki67 staining in tissues from miR-23b-3p knockdown and control mice. Compared with control mice, decreased expression of Ki67 (upper panel) were observed. Scale bars = 50 μm

First, we tested the expression level of miR-23b-3p in human OS cell lines. Four human OS cell lines, MNNG-HOS, U-2OS, MG63, and Saos-2, as well as one line of human osteoblast cell, hFOB1.19, were tested via quantitative real-time PCR. As shown in Fig. 1a, miR-23b-3p expression level in the OS cell lines was significantly higher than that in the osteoblast cell line. To further investigate the potential function of miR-23b-3p in OS cells, we constructed miR-23b-3p stable knockdown OS cell lines (MNNG-HOS and MG63) via short hairpin RNA (shRNA) method (Fig. 1b). First, we tested whether the knockdown of miR-23b-3p affected the proliferation of OS in vitro. Cell counting kit-8 (CCK-8) assays showed that downregulating miR-23b-3p significantly inhibited cell proliferation of MNNG-HOS and MG63 cells (Fig. 1c, d). Furthermore, compared with the control cells, knockdown of miR-23b-3p also reduced the number of cell colonies of OS cells (Fig. 1e). In addition, we evaluated the cell cycle distribution of miR-23b-3p-knockdown MNNG-HOS and MG63 cells, and found that the G1-phase population was increased, whereas the S-phase population was decreased compared with their negative control (NC) cells using flow cytometry (Fig. 1f). To explore the effects of miR-23b-3p on proliferation of OS cells in vivo, we established mouse xenograft models by injecting the control MNNG-HOS cells, as well as stable miR-23b-3p-knockdown MNNG-HOS cells into nude mice subcutaneously. The tumors with miR-23b-3p knockdown displayed an obvious delay in growth (Fig. 1f–i). In addition, immunohistochemical staining also showed a reduced expression of Ki67 in the miR-23b-3p-knockdown tumors (Fig. 1j). These findings suggested that miR-23b-3p promoted the proliferation of OS cells.

### miR-23b-3p inhibits OXPHOS and facilitates aerobic glycolysis in OS

**Fig. 2:**
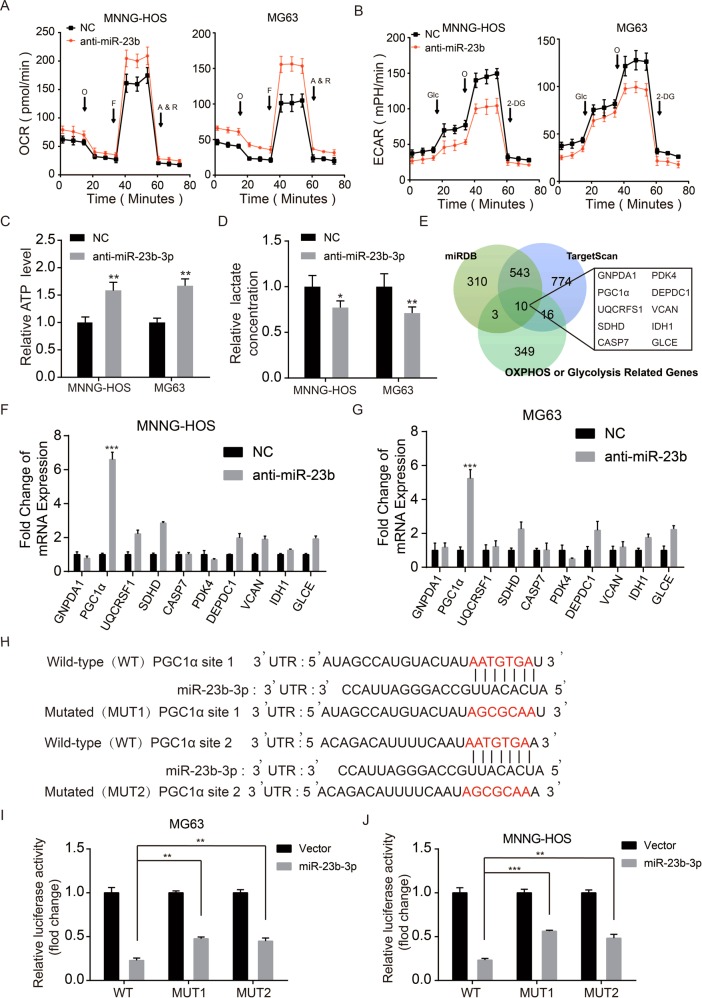
miR-23b-3p inhibited OXPHOS and facilitated the aerobic glycolysis in OS cells and directly targeted PGC1α. **a** O_2_ consumption rate (OCR) of MNNG-HOS and MG63 cells in control and miR-23b-3p kncokdown group was detected via a Seahorse Bioscience XFp analyzer. Glc: glucose, Oligo: oligomycin, 2-DG: 2-deoxy-d-glucose. **b** Extracellular acidification rate (ECAR) of MNNG-HOS and MG63 cells in control and miR-23b-3p knockdown group was detected using a Seahorse Bioscience XFp analyzer. O: Oligomycin, F: FCCP, A&R: antimycin A/rotenone. **c**, **d**. ATP level and lactate production was determined in MNNG-HOS and U-2OS cells stable knockdown miR-23b-3p. Values are means ± SD, *p < 0.05, **p < 0.01. **e** Venn diagram showing the predicted glycolysis or oxidative phosphorylation (OXPHOS)-related target genes of miR-23b-3p from databases (miRDB and TargetScan). **f**, **g**. The mRNA expression patterns of the predicted target genes of miR-23b-3p were determined by qRT-PCR in anti-miR-23b or Control MNNG-HOS and MG63 cells. **h** The wild-type and the mutated sequences of the PGC1α mRNA 3′-UTR (mutation site: red). **i**, **j**. The luciferase activity of the OS cells (MNNG-HOS and MG63) in luciferase reporter plasmid containing wild-type PGC1α 3′-UTR (Wt) and mutant PGC1α 3′-UTR (Mut1 and Mut2) co-transfected with miR-23b-3p mimics or a negative control was assessed, **p < 0.01, ***p < 0.001

There is no doubt that metabolic shift from OXPHOS to glycolysis promotes rapid proliferation of cancer cells^13^. To assess the effect of miR-23b-3p on glycolytic and mitochondrial flux in OS cells, we initially measured oxygen consumption rate (OCR, a marker of OXPHOS) and extracellular acidification rate (ECAR, a marker of glycolysis) of OS cells by using a seahorse 96XF Extracellular Flux Analyser. As shown in Fig. 2a, knockdown of miR-23b-3p enhanced both basal and maximal OCR in OS cells. In contrast, the basal and maximal ECAR were reduced when miR-23b-3p was silenced in OS cells (Fig. 2b). In addition, knockdown of miR-23b-3p in OS cells also elevated the formation of ATP produced by OXPHOS and reduced the level of lactate produced by the glycolysis (Fig. 2c, d). Collectively, these results demonstrated that miR-23b-3p inhibited OXPHOS and promoted aerobic glycolysis in OS cells.

### miR-23b-3p directly targeted PGC1α in OS cells

To explore the underlying mechanisms through which miR-23b-3p exerts its functional effects in OS cells, we predicted potential targets using two commonly used prediction algorithms TargetScan and miRDB. We found ten glycolysis or OXPHOS-related potential targets genes of miR-23b-3p (Fig. 2e). We compared the mRNA level of potential target genes between control group and miR-23b-3p-knockdown group in MNNG-HOS and MG63 cells. As revealed in Fig. 2f, g, knockdown of miR-23b-3p significantly suppressed mRNA expression of PGC1α. Furthermore, previous study had also predicted PGC1α as a potential target of miR-23^15^. The potential target sites of miR‑23b-3p on PGC1α was shown in Fig. 2h. To further confirm that miR-23b-3p could directly target PGC1α, dual luciferase reporter assays were performed. As shown in Fig. 2i, j, miR-23b-3p mimic led to a remarkable decrease in luciferase activity of wild-type PGC1α 3′-UTR (WT) reporter, but had no obvious effect on the luciferase activity in Mut 3′-UTR of PGC1α reporter in MG63 and MNNG-HOS cells. Taken together, our results indicated that PGC1α is a direct target of miR-23b-3p in OS cells.

### PGC1α promoted anti-Warburg effect and suppressed the proliferation of OS cells

**Fig. 3:**
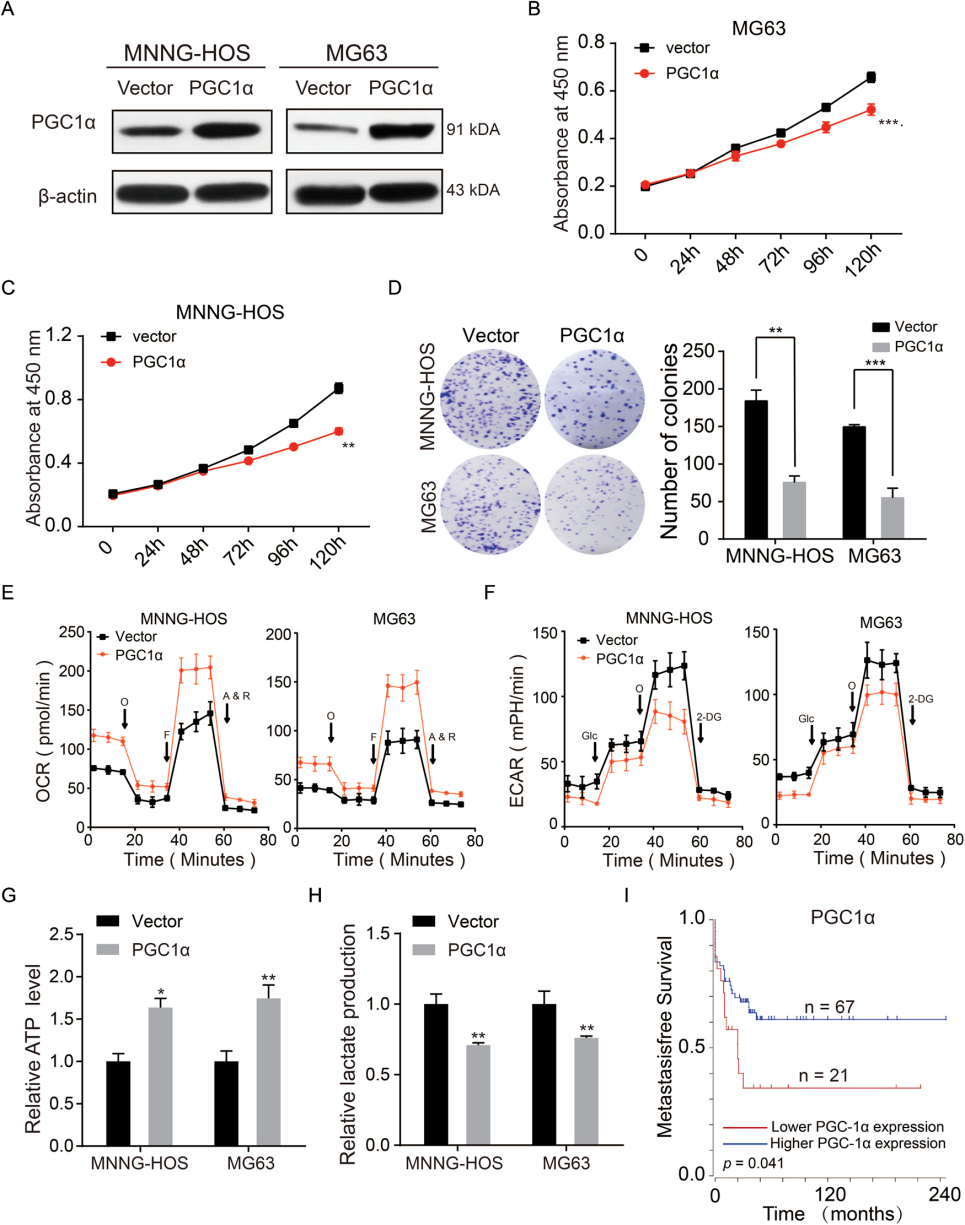
PGC1α inhibited OS proliferation and elicited anti-Warburg effect in OS cells. **a** Overexpression efficacy of PGC1α in OS cells (MNNG-HOS and MG63) was detected by western blotting. **b**, **c** Overexpression of PGC1α inhibited MNNG-HOS and MG63 cell proliferation using the cell counting kit (CCK)-8 assay. Values are means ± SD, **p < 0.01, ***p < 0.001. **d** Upregulation of PGC1α suppressed MNNG-HOS and MG63 cell proliferation using colony-formation assay. Values are means ± SD, **p < 0.01, ***p < 0.001. **e**, **f** OCR and ECAR of MNNG-HOS and MG63 cells in control and ov-PGC1α group were detected. **g**, **h** ATP level and lactate production was determined in MNNG-HOS and U-2OS cells stable overexpression PGC1α. Values are means ± SD, *p < 0.05, **p < 0.01. **i** Kaplan–Meier analysis of metastasis-free survival rate related to the expression of PGC1α expression in 88 OS cases based on a human osteosarcoma gene expression database. (https://hgserver1.amc.nl/cgi-bin/r2/main.cgi)

PGC1α was an indicator of mitochondrial biogenesis and OXPHOS, and had been reported to inhibit the growth of a number of cancers, such as hepatocellular carcinoma, renal cell carcinoma, malignant fibrous histiocytoma, and lung cancer^9,16–18^. However, the function of PGC1α in OS remains unclear. To explore the potential function of PGC1α in OS cells, we constructed PGC1α-stable overexpression of MG63 and MNNG-HOS cell lines (Fig. 3a). The results of CCK-8 assay and colony-formation assay indicated that upregulation of PGC1α remarkably inhibited the proliferation of OS (Fig. 3b–d). Upregulation of PGC1α also promoted the OXPHOS and inhibited aerobic glycolysis in OS cells (Fig. 3e, f). Furthermore, overexpression of PGC1α also elevated the formation of ATP produced by OXPHOS and reduced the level of lactate produced by the glycolysis (Fig. 3g, h). The prognostic significance of PGC1α in patients with OS was determined by using Kaplan–Meier analysis. Data on PGC1α were obtained from 88 OS patients from the R2 database. As shown in Fig. 3i, high PGC1α expression contributed to a better metastasis-free survival rate in OS patients. These data illustrated that PGC1α inhibited the proliferation of OS through promoting the switch of aerobic glycolysis to OXPHOS.

### Knockdown of PGC1α reversed the promotion on OXPHOS and inhibition on aerobic glycolysis and cell proliferation caused by miR-23b-3p knockdown

**Fig. 4:**
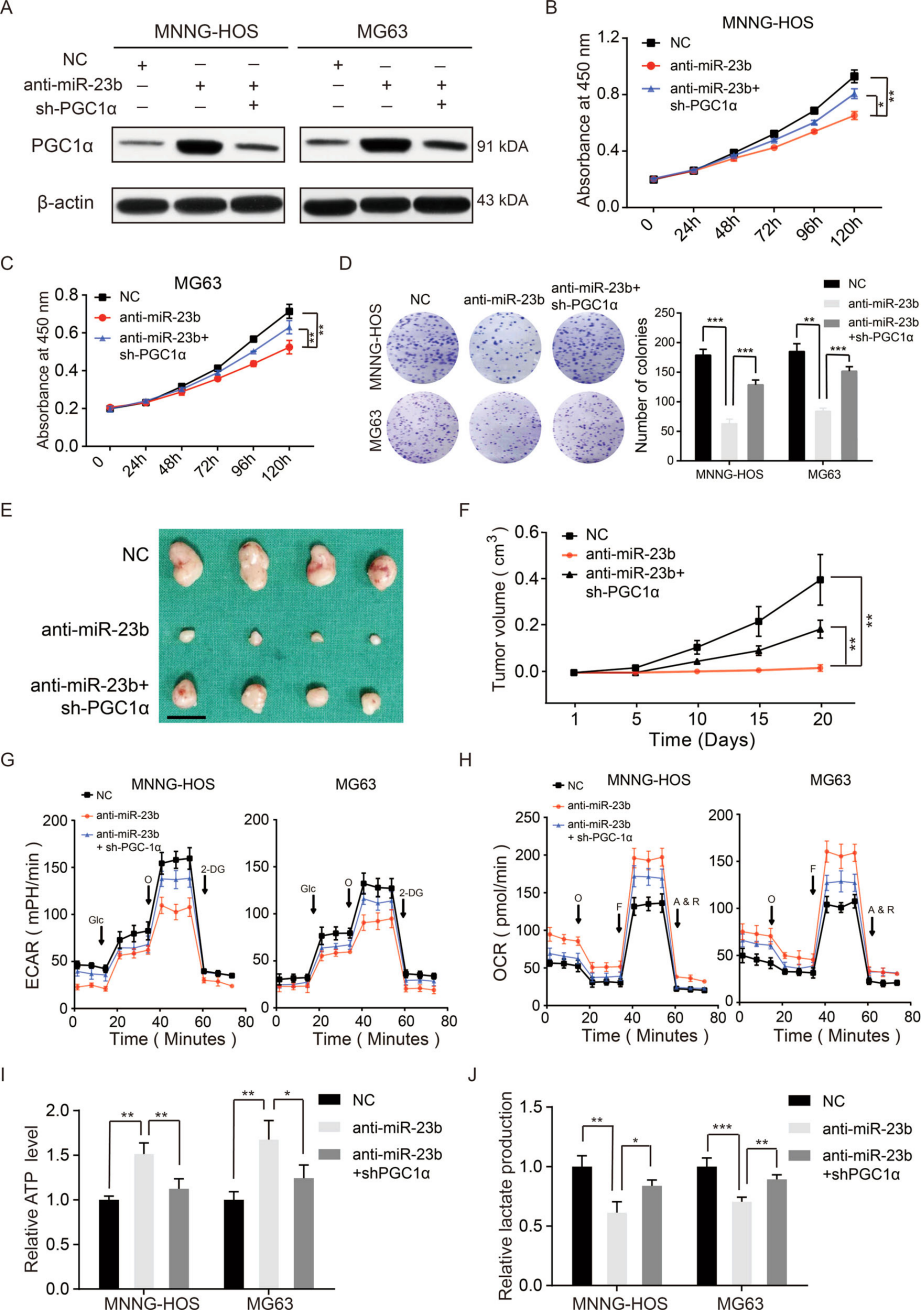
Knockdown of PGC1α partly rescued the effect of knockdown of miR-23b in OS. **a** Knockdown efficacy of PGC1α in miR-23b-3p knockdown MNNG-HOS and MG63 cells were determined by western blotting. **b**, **c** PGC1α knockdown partly reversed the inhibitory effects of miR-23b-3p knockdown on the colony-formation properties of OS (MNNG-HOS and MG63) cells, values are means ± SD, **p < 0.01, ***p < 0.001. **d** Silencing of PGC1α partly reversed the inhibitory effects of miR-23b-3p knockdown on the CCK-8 assay of MNNG-HOS and MG63 cells, values are means ± SD, *p < 0.05, **p < 0.01. **e** PGC1α knockdown partly reversed the inhibitory effects of miR-23b-3p silencing on the proliferation properties of MNNG-HOS cells. Excised tumors from different groups are shown (*n* = 4). Scale bars = 1 cm. **f** PGC1α knockdown partly rescued the inhibitory effects of miR-23b-3p knockdown on the growth rate in MNNG-HOS cells in vivo. Tumor volumes were measured with calipers every 5 days, values are means ± SD, **p < 0.01. **g**, **h** Altered level of OCR and ECAR in OS cells (MNNG-HOS and MG63) in different groups (Control, knockdown-miR-23b-3p, knockdown-miR-23b-3p, and knockdown- PGC1α). Values are means ± SD. **i**, **j**. ATP level and lactate production were determined in different groups (Control, knockdown-miR-23b-3p, knockdown-miR-23b-3p, and knockdown-PGC1α). Values are means ± SD, *p < 0.05, **p < 0.01, ***p < 0.001

We had demonstrated that miR-23b-3p promoted the proliferation and aerobic glycolysis of OS cells (Fig. 1); thus, we investigated whether the phenomenon regulated by miR-23b-3p was relied on its target PGC1α. We first downregulated miR-23b-3p together with PGC1α in MNNG-HOS and MG63 cell lines (Fig. 4a). As shown in Fig. 4b–d, knockdown of PGC1α diminished the inhibitory effect of miR-23b-3p knockdown on OS cells growth in vitro. In the in vivo assays, a compromised tumorigenic potential in miR-23b-3p knockdown group was partly offset via the downregulation of PGC1α (Fig. 4e, f). Similarly, the knockdown of PGC1α partly rescued the aerobic glycolysis in MNNG-HOS and MG63 cells (Fig. 4g, j). Taken together, these data suggested that miR-23b-3p promotes aerobic glycolysis and tumor growth by directly targeting PGC1α.

## Discussion

Recently, miR-23b-3p has been specifically linked to various functions in different cancers through a cell-type-dependent manner. In several kinds of tumors, miR-23b-3p was reported to act as a tumor inhibitor. Huang et al.^19^ showed that downregulation of miR-23b-3p in gastric carcinoma inhibited growth and invasion of gastric cancer cells through targeting Notch 2 and inhibiting its expression. miR-23b-3p inhibited tumorigenesis of prostate cancer by repressing Src kinase^20^. Moreover, miR-23b-3p acted as a tumor inhibitor by targeting Zeb1 in bladder cancer^21^.

However, miR-23b-3p was also related to the poor prognosis and acted as a cancer promoter in a number of tumors. Hu et al.^9^ introduced the hypothesis that miR-23b-3p functions as anti-apoptotic factor in gastric cancer cells by directly targeting Programmed cell death protein 4, an apoptosis regulatory protein. In breast cancer cells, miR-23b-3p negatively regulates Nischarin, an intracellular protein that acts as a tumor suppressor by regulating the metastatic behavior of tumor cells^10^. Moreover, Zaman et al.^17^ showed that upregulation of miR-23b promoted the migration of renal carcinoma cells through targeting Phosphatase and Tensin Homolog (PTEN). Here we demonstrated that miR-23b-3p was upregulated in OS cell lines and promoted the growth of OS cell lines (MNNG-HOS and MG63). Different from our findings, Liu et al.^22^ reported that miR-23b-3p inhibited the proliferation of OS cell line (U-2OS) through targeting SIX1 and inhibiting its expression, as it is well acknowledged that OS have multiple rearrangements across the genome, kataegis, and a high degree of intra- and intertumor heterogeneity^23–25^. The study of Christopher et al.^26^ also highlighted heterogeneity in growth rates and genetics among OS cell lines. Thus, the difference between these results may be due to a high-degree heterogeneity between OS cell lines. More OS cell lines would be used in future studies to further explore the biological functions of miR-23b-3p in OS.

A glucose metabolism characteristic of most cancer cells is the favor of glycolysis for ATP production rather than OXPHOS even when oxygen is abundant, which also was called Warburg effect^27^. There was no doubt that enhanced aerobic glycolysis or Warburg effect promoted rapid proliferation in cancer cells through increasing biosynthesis of macromolecules, enhancing disruption of tissue architecture and immune cell evasion, and so on^28^. Thus, insights into this characteristic alteration and promoting a switch from aerobic glycolysis to OXPHOS will hold promise for potential cancer therapy^29^. However, little is known about the role of miRNA in glucose metabolism reprogramming of OS. Here we reported that miR-23b-3p acts as a novel promoter of Warburg effect by targeting PGC1α directly and inhibiting its expression in OS.

OXPHOS dysfunction is known to lead to compensatory glycolysis^30^. It has been reported that upregulation of PGC1α could facilitate OXPHOS and inhibit aerobic glycolysis, which is also called the anti-Warburg effect, in several types of tumors^13,14^. Furthermore, PGC1α has been reported to have an anti-tumorigenic function in OS. Yasuo et al.^31^ reported that PGC1α was downregulated in OS tissue and overexpression of PGC1α could promote the apoptosis of OS cells. In this study, we reveled a new mechanism that upregulation of miR-23b-3p inhibited the expression of PGC1α by directly targeting it and led to a suppression of OXPHOS and a promotion of aerobic glycolysis in OS cells.

In summary, our study revealed the role and mechanism of miR-23b-3p in the OS proliferation by regulating glucose metabolism reprogram. The upregulation of miR-23b-3p inhibited OXPHOS and promoted aerobic glycolysis through targeting PGC1α and inhibiting its expression in OS. Consequently, targeting the miR-23b-3p/ PGC1α axis may be helpful to provide new anti-cancer strategy for OS patients.

## Materials and methods

### Reagents

Antibodies against PGC1α (ab54481; Abcam, Cambridge, UK), β-actin (ab8227; Abcam, Cambridge, UK), and Ki67 (ab15580; Abcam, Cambridge, UK) were used.

### Cell lines and culture

The human OS cell lines U-2OS, MNNG-HOS, Saos-2, and MG63, and human osteoblast cell line hFOB1.19 were purchased from the Cell Bank of Shanghai Institute of Biological Science (Shanghai, China). MNNG-HOS and MG63 were incubated with Eagle’s minimum essential medium (Gibco, USA), whereas Saos-2 and U-2OS were incubated with McCoy’s 5a Medium (Gibco, USA) containing 10% fetal bovine serum at 37 °C in the presence of 5% CO_2_. hFOB1.19 were incubated with F12 Medium (Gibco, USA) at 34.5 °C in the presence of 5% CO_2_.

### Plasmid transfection

Lentiviral vectors expressing PGC1α, miR‑23b-3p shRNA, PGC1α shRNA, and control construct were purchased from GenePharma (Shanghai, China). Stable cell lines overexpressing PGC1α, PGC1α shRNA, or/and miR‑23b-3p shRNA were established by infection with the lentiviruses respectively with polybrene (GM-040901B, Genomeditech, Shanghai, China) according to the manufacturer’s instructions. The infected cells were selected using puromycin or blasticidin. miR-23b-3p mimics were purchased from GenePharma (Shanghai, China).

### RNA extraction and quantitative real-time PCR

Trizol reagent (Sigma, USA) was used to extract total cellular RNA containing miRNA from cultured cells. SYBR® Premix Ex Taq™ kit (Takara Bio, Japan) was used to quantify the transcripts of target mRNA via the Applied Biosystems 7500 Real-Time PCR system (Thermo Fisher Scientific, Inc.). Target miRNA was reverse transcribed to cDNA and expression level was determined through a miRNA qRT-PCR Sybgreen Detection Kity (A2030A003, Bio-TNT Biotechnologies, China) according to the manufacturer’s instructions. The reaction was performed with one cycle of 95 °C for 5 min and 40 cycles of 95 °C for 5 s, 60 °C for 30 s using the Applied Biosystems 7500 Real-Time PCR system (Thermo Fisher Scientific, Inc.). 18S and U6 were amplified as an internal reference.

### Western blotting

Total cellular proteins were extracted from the target cells by RIPA lysis buffer (Beyotime, Shanghai, China) according to the manufacturer’s instructions. Equal amounts of proteins were loaded onto 10% Tris-glycine SDS-polyacrylimide gel electrophoresis gels (Bio-Rad Laboratories, CA, USA). Then the separated proteins were transferred onto nitrocellulose membranes (Millipore, MA, USA). After blocking with 5% non-fat milk, the membranes were incubated with a primary antibody at 4 °C overnight. The membranes were further incubated with secondary antibody and protein signals was detected under the ECL detection kit (Share-bio, Shanghai, China).

### CCK-8 assay

Cell proliferation was measured by a CCK-8 kit (CK04, Dojindo Molecular Technologies, Kumamoto, Japan) according to the manufacturer’s instructions. Cells were inoculated into 96-well plates (3 × 103 cells/well) and then added 10 μL of CCK-8 reagent into each well at 0, 24, 48, 96, and 120 h. Absorbance at 450 nm was measured using a spectrophotometric plate reader. Each experiment was done in triplicate for three times.

### Colony-formation assays

Target cells were seeded at a density of 1000/well into 6-well plate. Two weeks later, colonies were washed with phosphate-buffered saline (PBS) twice and fixed with 4% paraformaldehyde. At the end, colonies were stained by crystal violet and counted.

### Cell cycle analysis

Target cells were collected and washed with cold PBS and fixed in ice-cold 70% ethanol at 4 °C overnight. The target cells were recovered by centrifugation at 800 r.p.m. for 5 min and washed with PBS for 10 min. Then, 1 ml DNA staining solution and 10 µl permeabilization solution (MultiSciences Biotech Co., HangZhou, China) were added and the cells were incubated for 30 min in dark. Cell cycle was determined and analyzed by flow cytometry through using the BD FACSCalibur platform.

### Mouse xenograft model

BALB/c nude mice (4–6 weeks old, male) were obtained from East China Normal University. All animal experiments and animal care were conducted following the Guide for the Care and Use of Laboratory Animals, Institute of Laboratory Animal Resources. MNNG-HOS cells (1.5 × 10^6^) with stable miR-23b-3p knockdown or stable PGC1α overexpression, or control plasmid re-suspended in 200 μl PBS were subcutaneously injected the nude mice. Tumor size and body weight of each nude mouse was measured every 5 days over a period of 20 day. On day 20, the mice were killed and tumor tissues were collected, fixed, and prepared for further analysis. The volume of tumor was calculated through the following equation: tumor volume = length × width^2^/2.

### Measurement of glucose metabolism

Glucose flux in mitochondrial and glycolysis was measured using the Seahorse XF-96 metabolic flux analyser (Seahorse Bioscience, North Billerica, MA, USA) according to the manufacturer’s instructions. OCR and ECAR were measured using Seahorse XF Cell Mito Stress Test Kit and Seahorse XF Glycolysis Stress Test Kit, respectively. Briefly, 3 × 10^4^ target cells were seeded into each well of a Seahorse XF-96 cell culture microplate. After the probes were calibrated, for OCR, 1 mM oligomycin, 1 mM p-trifluoromethoxy carbonyl cyanide phenylhydrazone and 2 mM antimycin A plus 2 mM rotenone (Rote/AA) were sequentially injected, and for ECAR, 10 mM glucose, 1 mM oligomycin, and 80 mM 2-deoxyglucose were sequentially injected. Data were assessed and analyzed by using Seahorse XF-96 Wave software.

### Measurement of ATP level and lactate production

Cellular ATP levels were determined by using a luminescence ATP detection kit (Promega, Madison, WI) according to the manufacturer’s protocol. Luminescence signals was measured by using a plate reader (PerkinElmer, Waltham, MA). ATP levels were calculated from standard curves. Extracellular lactate was detected using the lactate assay kit (BioVision, CA, USA) according to the manufacturer’s instructions. All values were normalized to cellular protein level.

### Dual luciferase reporter assay

For luciferase activity analysis, 1 × 10^5^ MNNG-HOS or U-2OS cells were planted into each well of 96-well plates the day before transfection. The cells were transiently transfected with WT PGC1α 3′-UTR or mutant PGC1α 3′-UTR were cloned into the p-MIR-reporter plasmid, combined with either miR-23b-3p mimics or NC using Lipofectamine 2000 (Invitrogen, Carlsbad, CA, USA). Forty-eight hours later, luciferase activity was measured with a Dual-Luciferase Assay System (Promega, Madison, WI, USA).

### Database analysis

Prognostic analysis of PGC1α expression for OS patient survival was performed by using R2 database. The data from TargetScan and miRDB were used to analyze potential target genes of miR-23b-3p.

### Statistical analysis

The data were expressed as the mean ± SD. Prism 5.0 (GraphPad, CA, USA) was used for data statistical analyses. Differences between the control and treated groups were analyzed by using two-tailed Student’s *t*-test. The differences were considered statistically significant at *p* < 0.05 (*p < 0.05; **p < 0.01; ***p < 0.001).
